# Programme of the American Anti-Tuberculosis League for the Prevention of Consumption

**Published:** 1905-03

**Authors:** 


### PROGRAM OF THE AMERICAN ANTI-TUBERCULOSIS LEAGUE FOR THE PREVENTION OF CONSUMPTION.

---

MEETING HALL OF HOUSE OF REPRESENTATIVES GEORGIA STATE CAPITOL, ATLANTA, GA., U. S. A., APRIL 17–19, 1905.

HONORARY PRESIDENTS—Dr. J. Riviere, Dr. Guilliam Livet, Paris, France.

PRESIDENT AND EXECUTIVE OFFICER—Dr. George Brown, Atlanta, Ga.

VICE-PRESIDENTS—Drs. H. H. Stone, Phœnix, Arz.; Seale Harris, Union Springs, Ala.; S. E. Gavan, Fon du Lac, Wis.; George R. Dean, Spartanburg, S. C.; J. H. Walker, Effingham, Ill.; C. Barlow, Robinson, Ill.; W. S. Alexander, Oxford, O.; L. P. Hammond, Rome, Ga.; J. W. Beach, Pittsburg, Pa.; F. S. Twitty, Columbia, Ala.; J. P. Loyd, Shreveport, La.; Cassius T. Lessan, Mt. Ayr, Iowa; R. H. Tatum, Chattanooga,

Tenn.; S. W. Pryor, Chester, S. C.; Frank C. Wilson, Louisville, Ky.; G. H. Cattermole, Boulder, Colo.

EXECUTIVE COMMITTEE — Drs. J. H. Hallock, Chairman, Saranac Lake, N. Y.; W. Pennell, Mt. Vernon, Ohio; C. D. Morgan, Galoin, Ohio; Mark Millikin, Hamilton, Ohio; Franklin Smith, New York City; J. H. Demarest, New York City; J. L. Walker, Waycross, Ga.; A. H. VanDyke, Atlanta, Ga.; T. V. Hubbard, Atlanta, Ga.

COMMITTEE ON SANITORIA — Dr. T. W. Seward, Chairman, Las Vegas, N. M.

COMMITTEE ON ELECTRO-THERAPEUTICS — Dr. J. Rudis-Jicinsky, Chairman, Cedar Rapids, Iowa.

(Other committees to be appointed.)

ATLANTA, GA., February 1, 1905.

*To The Medical Profession:*

The next meeting of the American Anti-Tuberculosis League will be held in Atlanta, Ga,, April 17th to 19th, 1905.

Governor J. M. Terrell has tendered the Hall of the House of Representatives in the Georgia State Capitol for the use of the League during the meeting. He will deliver an address to the League on the first morning, as will other distinguished men.

The opening session is intended to be a broad one, in an educational sense, and the heads of the largest educational institutions of the United States will be invited to be present.

Reduced rates will be had on all roads. Hotel rates will also be made special for visitors. Over one thousand delegates representing the following leading National and State medical societies have been enrolled:

American Public Health Association, appointed by Dr. Henry Holton, President; Georgia State Medical Association, appointed by Dr. Charles Hicks, President; Connecticut Medical Society, appointed by Dr. S. H. Shelton, President; Arkansas Medical Society, appointed by Dr. Leonidas Kirby, President; Louisiana State Medical Society, appointed by Dr. J. M. Barrier, President; Tennessee State Medical Association, appointed by Dr. I. A. McSwain, President; Alabama State Medical Association, appointed by Dr. M. B. Cameron, President; American Gynecological Society, appointed by Dr. J. E. Janvrin, President; Colorado State Medical Society, appointed by Dr. W. W. Grant, President; Iowa Health Officers' and City Physicians' Association, appointed by Dr. J. A. Valenta, President; South Carolina Medical Association, appointed by Dr. E. F. Darby, President; The Association of Military Surgeons, appointed by Dr. John C. Wise, President; Mississippi Valley Medical Association, appointed by Dr. Edwin Walker, President; Maine Medical Society, appointed by Dr. Thayer, President; Arizona Medical Association, appointed by Dr. L. D. Dameron, President; Kentucky State Medical Association, appointed by Dr. Steele Bailey, President; Tri-State Medical Society of Alabama, Georgia and Tennessee, appointed by Dr. Michael Hoke, President; South Dakota State Medical Association, appointed by Dr. B. A. Babb, President; Northwestern Pennsylvania Medical Society, appointed by Dr. C. W. Coulten, President; North Dakota State Medical Society, appointed by Dr. W. H. Bodenstah, President; Medical Society of the State of North Carolina, appointed by Dr. H. B. Weaver, President; National Association of United States Pension Examining Surgeons, appointed by Dr. Wm. A. Howe, President; Association of Railway Surgeons of the Pacific Slope, appointed by Dr. Walter B. Coffee, President; New Hampshire Medical Society, appointed by Dr. Ezra Mitchell, President; Fox River Valley Medical Association, appointed by Dr. G. J. Schneider, President; Ohio State Pediatric Society, appointed by Dr. F. W. Mitchell, President: American Protologic Society, appointed by Dr. Wm. M. Beach, President; New Hampshire Association of Boards of Health, appointed by Dr. Irving A. Watson, President; Illinois State Medical Society, appointed by Dr. Carl E. Black, President; American Electric-Therapeutic Association, appointed by Dr. Alfonso David Rockwell, President; Arkansas State Homœpathic Medical Association, appointed by Dr. W. E. Green, President; Rhode Island Medical Society, appointed by Dr. Stephen A. Welch, President; American Pediatric Society, appointed by Dr. Augustus Caille, President; National Electric Medical Association, appointed by Dr. Rolla L. Thomas, President; Maine Academy of Medicine and Science, appointed by Dr. Daniel Driscsel, President; Pacific Coast Association of Insurance Examiners, appointed by Dr. Wm. F. Amos, President; The Anglo-American Medical Association of Berlin, Germany, appointed by Dr. J. Chester King, President; American Institute of Homœopathy, appointed by Dr. John P. Sutherland, President; C. H. & D. Railway Surgeon Association, appointed by Dr. W. S. Hoy, President, and numerous other county and State medical associations.

Papers have been promised for this meeting of unusual interest by Doctors J. Riviere and Guilliam Livet, of Paris. The latter giving a new treatment for consumption which has been tested in his clinic for the past two years and has never before been published.

Papers by the most distinguished men in the United States have been promised.

The Atlanta Chamber of Commerce will tender a reception to the visiting members.

A ladies' reception committee, of which Mrs. T. M. Horner is chairman, will tender a reception to visitors and see that their wives and families are cared for.

The following committee of local physicians will look after the comfort of the visitors: Drs. A. H. VanDyke, Chairman; W. S. Armstrong, S. T. Barnett, W. E. Campbell, W. L. Champion, J. D. Cromer, J. G. Earnest, A. L. Fowler, W. S. Goldsmith, T. V. Hubbard, G. P. Huguley, J. C. King, T. C. Longino, Edgar G. Ballenger, T. H. Hancock, W. C. Jernigan, E. Van Goitsnoven, E. C. Davis, E. L. Griffin, S. G. C. Pinckney, W. P. Nicholson, J. C. Olmstead, L. P. Stevens, C. W. Stickler, W. F. Westmoreland, B. Wolff, H. S. Wright, J. D. Thompson, T. D. Longino, M. B. Hutchins, E. P. Flowers, W. T. Asher, H. R. Donaldson, McFadden Gaston, Joe Bradfield, W. T. Brown, J. B. S. Holmes, E. H. Richardson, J. N. Ellis.

We earnestly request every member of the medical profession of the United States who is interested in the prevention of tuberculosis and who has the good of humanity at heart, to be with us and help us with this great work which we have started so auspiciously and for which there is such a great field for suffering humanity.

Very truly yours,

GEO. BROWN,

President and *ex officio.*

The following bodies will meet at the same time and place :

GEORGIA STATE COMMISSION ON TUBERCULOSIS.—Drs. Chas. Hicks, Chairman, Dublin; Bernard Wolff, Permanent Secretary, Atlanta.

MEMBERS FROM STATE AT LARGE.—Drs. Chas. Hicks, Dublin; Louis H. Jones, Atlanta; Ernest P. Ham, Gainesville; P. L. Hilsman, Albany; Jos. R. Burdette, Tennille; Pryor W. Fitts, Greenville; L. G. Hardman, Commerce; L. J. Belt, Millen; J. L. Walker, Waycross; S. A. Brown, Dalton.

MEMBERS FROM CONGRESSIONAL DISTRICT.—Drs. E. R. Corson, Savannah; O. B. Bush, Camilla; Alex. Mack, Hawkinsville; H. R. Slack, LaGrange; Bernard Wolff, Atlanta; George F. Alexander, Forsyth; Thomas R. Garlington, Rome; Henry E. Thornton, Hartwell; Jefferson Davis, Toccoa; Thomas D. Coleman, Augusta; S. E. Sanchez, Barwick.

THE GEORGIA ANTI-TUBERCULOSIS LEAGUE.—Dr. Willis F. Westmoreland, President, Atlanta, Ga.; Dr. T. Virgil Hubbard, Secretary, Atlanta, Ga.

VICE-PRESIDENTS.—Drs. R. M. Harbin, Rome, Ga.; Samuel C. Benedict, Athens, Ga.; H. L. Gill, Columbus, Ga.; W. P. Taylor, Thomasville, Ga.; E. M. Coleman, Atlanta, Ga.; E. C. Davis, Atlanta, Ga.; W. B. Emery, Atlanta, Ga.; W. L. Dean, Woodstock, Ga.; R. L. Guinn, Conyers, Ga.; H. L. Carson, Griffin, Ga.; J. B. Morgan, Augusta, Ga.; C. T. Nolan, Marietta, Ga.; C. H. Peete, Macon, Ga.; H. L. Rudolph, Gainesville, Ga.; M. M. Saliha, Savannah, Ga.

